# Opinion Delivered by President Judge Hanna, Orphans' Court, Philadelphia, in the Waesch's Estate Case

**Published:** 1894-07

**Authors:** 


PRESIDENT JUDGE HANNA, in delivering the opinion of the Orphans' Court of Philadelphia, in the case of Waesch's Estate says: The liability of the husband for medical attendance upon his wife, even although she has a separate estate, is too well settled to admit of argument. He is primarily liable, and her estate is liable only in case he be insolvent. If any balance then remains for distribution, the expenses he should have paid will be deducted from his distributive share.

